# Graphene-oxide-microfiber aptasensor for dynamic neutrophil monitoring: Bridging cytokine release syndrome and point-of-care sepsis diagnosis

**DOI:** 10.1016/j.isci.2026.117823

**Published:** 2026-10-12

**Authors:** Zhiheng Yu, Heshuang Dong, Xiaolin Chen, Mengjiang Xu, Ren Liu, Jingjing Wang, Cunliang Yang, Ke Wang, Xianyuan Zhao, Hao Jia, Jijun Feng

**Affiliations:** 1 Shanghai Key Laboratory of Modern Optical System, Engineering Research Center of Optical Instrument and System (Ministry of Education), School of Optical-Electrical and Computer Engineering, University of Shanghai for Science and Technology, Shanghai 200093, China; 2 Faculty of Basic Medicine, Shanghai Jiao Tong University School of Medicine, Shanghai 200025, China; 3 Department of Critical Care Medicine, Renji Hospital, School of Medicine, Shanghai Jiao Tong University, Shanghai 200120, China; 4 QingPu Branch of Zhongshan Hospital Affiliated to Fudan University, Shanghai 201700, China

**Keywords:** neutrophil elastase, lactoferrin, graphene oxide, optical microfibers, early sepsis diagnosis

## Abstract

Neutrophil granule release is critical for host defense, yet its dysregulation underpins cytokine release syndrome (CRS) in both acute (such as sepsis) and sustained inflammatory states. Distinguishing these phases requires real-time monitoring of early markers like neutrophil elastase (ELANE) and lactoferrin (LF), but conventional assays lack requisite sensitivity. Here, we developed a graphene-oxide-functionalized optical microfiber biosensor with high-affinity DNA aptamers (NE-AP and LF-AP) to quantify ELANE and LF via aptamer-protein binding-induced spectral red shifts. The sensor achieved ultralow detection limits (3.57 × 10^−20^ M for ELANE and 1.28 × 10^−19^ M for LF), eight orders below ELISA, with excellent selectivity. In a lipopolysaccharide-induced murine model, it resolved distinct temporal release profiles of ELANE and LF, and in clinical validation, it showed distinct spectral responses between septic patients and healthy controls in a preliminary clinical cohort. This platform provides a highly sensitive, real-time tool for decoding neutrophil dynamics, with preliminary translational potential for early sepsis diagnosis.

## Highlights

• GO-microfiber aptamer biosensor enables ultrasensitive ELANE/LF protein detection
• Limits of detection is eight orders of magnitude lower than the conventional ELISA
• Dynamic monitoring of mouse inflammation model reveals biomarker release profiles
• Sensor robustly distinguishes septic patient from healthy controls in clinical validation

## Graphical abstract

**Figure:**
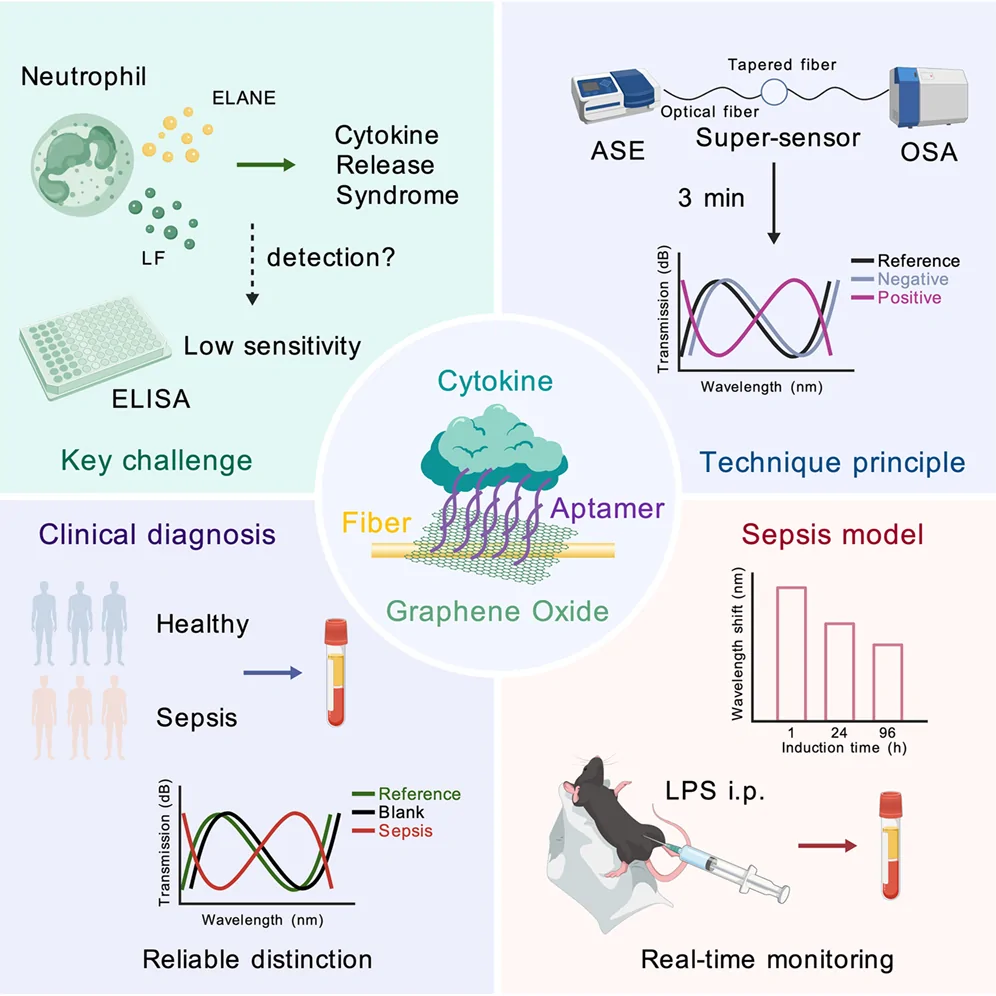

## Teaser

Biological sciences; Immunology; Cell biology

## Introduction

Neutrophils are the core effector cells of the innate immune system and play a pivotal role in infection and inflammatory responses.^1^^,^^2^^,^^3^ Upon pathogen invasion or tissue injury, neutrophils rapidly migrate to inflammatory sites and eliminate pathogens through degranulation, reactive oxygen species production, and neutrophil extracellular trap formation.^4^^,^^5^^,^^6^^,^^7^ However, excessive activation of neutrophils leads to the release of large amounts of inflammatory mediators (such as neutrophil elastase [ELANE] and lactoferrin [LF]),^8^^,^^9^ causing tissue damage and multi-organ dysfunction, which are closely associated with various diseases such as sepsis^10^ and chronic inflammation.^11^ For instance, sepsis patients exhibit significantly elevated neutrophil activation levels, which correlate strongly with disease severity and prognosis.^6^^,^^10^ Therefore, real-time monitoring of neutrophil activation status is crucial for early disease diagnosis, treatment monitoring, and prognosis evaluation.

Cytokine release syndrome (CRS) represents a common pathophysiological pathway underlying both acute and chronic inflammatory conditions, wherein dysregulated neutrophil activation leads to the excessive release of inflammatory mediators that cause systemic tissue damage and multi-organ dysfunction. CRS manifests across a clinical spectrum: at one extreme, the acute, life-threatening hyperinflammatory state characteristic of sepsis; at the other, the sustained, low-grade inflammatory milieu that drives chronic inflammatory diseases. Critically, the temporal evolution of neutrophil degranulation provides a molecular readout of CRS progression. ELANE, stored in primary (azurophilic) granules, is released rapidly within minutes to hours upon neutrophil activation, serving as an early indicator of acute systemic inflammation. In contrast, LF, sequestered in secondary (specific) granules, exhibits a delayed and more sustained release profile, reflecting ongoing or resolving inflammatory responses. The distinct release kinetics of these two granule components therefore offer complementary windows into the acute and sustained phases of CRS, making their simultaneous detection particularly valuable for differentiating inflammatory states and guiding timely clinical intervention. However, conventional detection methods have been unable to capture the early, low-abundance release events that precede fulminant CRS, underscoring the urgent need for ultrasensitive, real-time monitoring technologies.

Currently, neutrophil activation assays rely on flow cytometry^12^ and plasma marker assays. However, these methods have limitations including complex operation, low throughput, and insufficient sensitivity.^13^^,^^14^ Although plasma biomarker detection is relatively simple to perform, its sensitivity and specificity are constrained, and it cannot distinguish between neutrophil subpopulations with different activation states. Moreover, the performance of traditional methods in complex biological samples is often compromised by nonspecific adsorption and matrix effects.

In recent years, sensors based on molecular recognition technology have emerged as a research hotspot, though their application in neutrophil activation detection remains in its infancy. Aptamers, single-stranded DNA or RNA molecules selected through the systematic evolution of ligands by exponential enrichment (SELEX) process, exhibit high affinity and specificity for target molecules.^15^ Due to their ease of synthesis, stability, and high specificity, aptamers demonstrate considerable potential as bioreceptors in clinical diagnostics and therapeutics. In neutrophil activation detection, aptamer technology offers distinct advantages. For example, aptamers can specifically bind key effector molecules such as ELANE and LF, avoiding cross-reactivity issues common in traditional antibody-based methods.^16^ Furthermore, aptamer-based sensors enable real-time and dynamic monitoring, providing a novel tool for investigating neutrophil activation mechanisms. However, most of the existing aptamer sensors are based on electrochemical^17^^,^^18^ or fluorescence^19^ detection, and their sensitivity and anti-interference ability still need to be further improved. Significantly, a highly sensitive electrochemical aptamer sensor for organophosphorus detection via a one-step co-electrodeposition method for aptamer immobilization was developed by Fu et al.^20^ Meanwhile, this approach offers a simple and efficient preparation process, effectively addressing the complexity associated with conventional fabrication methods. A ratiometric electrochemical aptamer sensor by immobilizing the aptamer onto a screen-printed carbon electrode via gold nanoparticles and terminal thiol groups (forming Au–S bonds) was prepared by Xu et al.^21^ This method effectively addresses the issues of low accuracy and significant batch-to-batch variability encountered in conventional approaches. A highly sensitive dual-ratiometric electrochemical aptamer sensor by encapsulating ZIF-8 nanocomposites with gold nanoparticles was fabricated by Li et al.^22^ This sensor enables simultaneous detection of malathion and propiconazole, offering a promising approach for multi-target analysis in food safety and environmental monitoring applications. Although the sensitivity of electrochemical aptamer sensors has been significantly improved through various signal amplification strategies, they still face inherent challenges including susceptibility to electromagnetic interference in clinical settings, limited multiplexing capacity due to electrode cross-talk, and the need for complex sample pre-treatment. In contrast, optical microfiber sensors offer distinct advantages: label-free and real-time detection, inherent immunity to electromagnetic interference, potential for multi-parameter integration along a single fiber, and ease of miniaturization for point-of-care applications. These complementary strengths position optical microfiber platforms as a promising alternative for clinical biosensing.

Graphene oxide (GO), a novel two-dimensional nanomaterial, exhibits excellent biocompatibility and a high surface area, significantly enhancing sensor sensitivity and selectivity.^23^^,^^24^ Microfiber sensors, known for their high sensitivity, rapid response, and miniaturization, demonstrate great potential in biosensing applications.^25^^,^^26^ Liu et al. developed an optical microfiber sensor integrating DNA aptamers and GO for highly sensitive detection of tuberculosis antigens^25^ A fiber-optic near-field enhanced plasmonic resonance immunosensor for specific recognition of liver cancer biomarkers was designed by Jing et al.^27^ A GO-coated optical microfiber aptamer sensor was innovatively applied for real-time monitoring of SARS-CoV-2 nucleocapsid protein by Jia et al.^28^^,^^29^ Additionally, Lee et al. developed a label-free fiber-optic LSPR aptamer sensor for the quantitative detection of ochratoxin A^30^ A GO-based micro-tapered long-period fiber grating sensor was proposed by Kang et al.^31^ It offered a new approach for sensitive detection of pepsin.

In this paper, we developed a rapid neutrophil activation detection system based on a GO-microfiber sensor. The system immobilizes GO on an amino-functionalized optical microfiber surface and utilized electrostatic adsorption to immobilize the aptamers that specifically bind to ELANE and LF, enabling highly sensitive and specific detection of key neutrophil activation markers (ELANE and LF). The sensor requires only immersion of the probe into the sample solution, achieving limits of detection of 3.57 × 10^−20^ M for ELANE and 1.28 × 10^−19^ M for LF. With advantages such as simple operation and ease of miniaturization, this detection system provides a novel technological approach for early diagnosis of sepsis and chronic inflammation, demonstrating significant potential for clinical real-time monitoring and disease diagnostics.

## Results

### Expression and purification of ELANE and LF protein and selection of aptamers

The nucleotide sequences encoding ELANE and LF were cloned into an expression vector carrying a His-tag to facilitate protein purification. The specificity and purity of the recombinant proteins were verified by SDS-PAGE analysis (Figures 1A and 1F). The observed molecular weights of ELANE (52.6 kDa) and LF (77 kDa) were consistent with the values reported in previous studies, confirming the successful expression of the target proteins. We selected optimal aptamers (NE-AP for ELANE and LF-AP for LF) and predicted their secondary structures using NUPACK (Figures 1B and 1G). Furthermore, Using RNA Composer, we predicted the high-resolution three-dimensional structures of ELANE and LF (Figures 1C and 1H). Molecular docking analysis revealed the potential binding sites between ELANE and LF and their corresponding aptamers (Figures 1D and 1I), highlighting the strong and specific interactions between each aptamer and its target protein. These computational results provide structural insights into the molecular recognition mechanisms and establish a solid theoretical foundation for subsequent biosensing applications.

**Figure 1:**
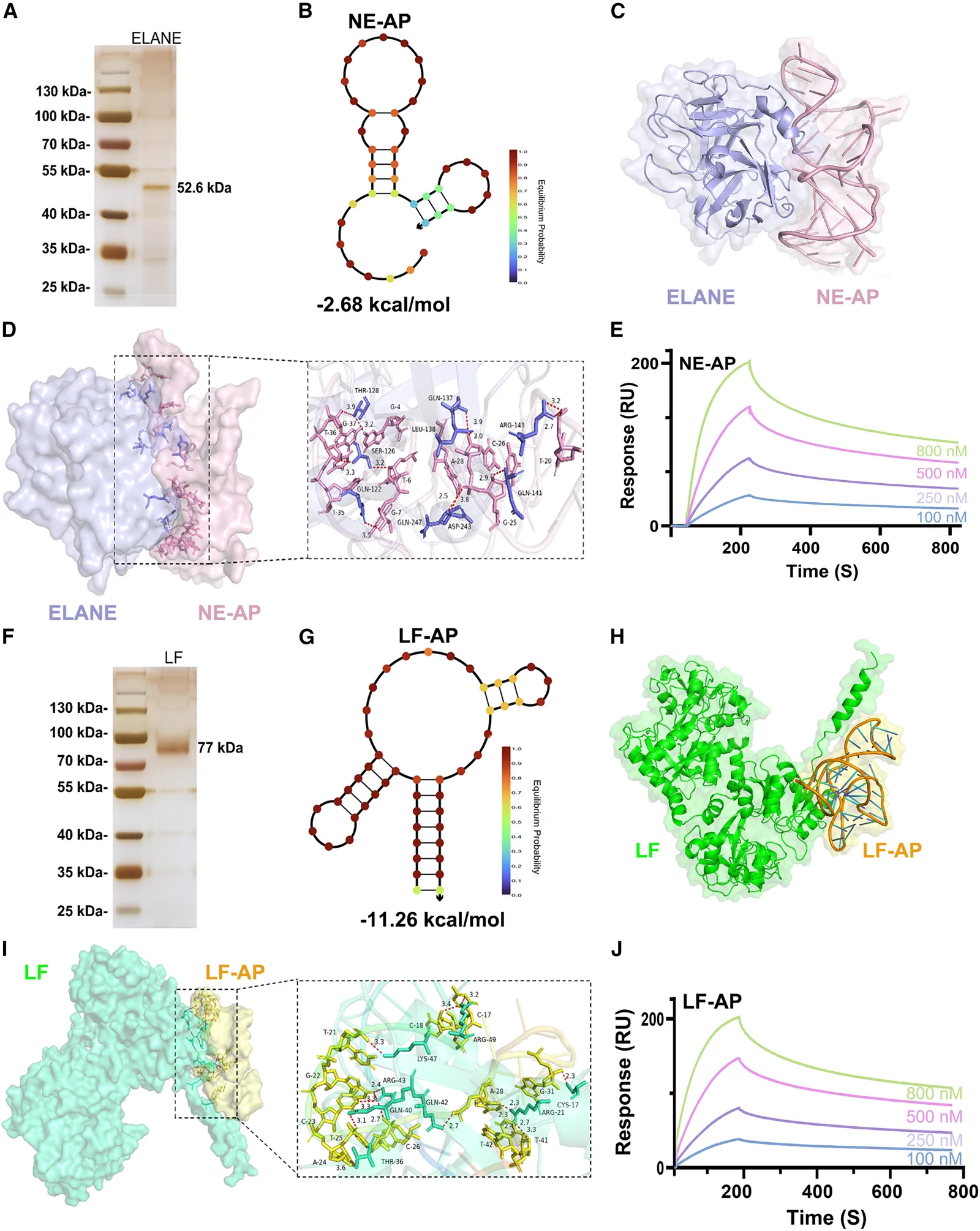
Characterization of ELANE- and LF-specific aptamers (A) Western blot analysis of purified ELANE protein by SDS-PAGE. (B) Sequence and predicted secondary structure of the NE-AP aptamer bound to ELANE protein. (C) Molecular docking simulation of the ELANE–NE-AP complex predicted using the HDOCK server and visualized with PyMOL. (D) Detailed interaction view of the ELANE-NE-AP docking complex generated by BioGDP.com. (E) Binding affinity measurement of NE-AP aptamer to ELANE protein. (F) Western blot analysis of purified LF protein by SDS-PAGE. (G) Sequence and predicted secondary structure of the LF-AP aptamer bound to LF protein. (H) Molecular docking simulation of the LF-LF-AP complex predicted using the HDOCK server and visualized with PyMOL. (I) Detailed interaction view of the LF-LF-AP docking complex generated by BioGDP.com. (J) Binding affinity measurement of LF-AP aptamer to LF protein. *n* = 3 independently expressed protein preparations (A and F). *n* = 3 independent flow cell injections per aptamer concentration on three independently prepared sensor chips (E and J).

### Specific binding of NE-AP and LF-AP to their corresponding proteins

The binding specificity and affinity of the ssDNA aptamers (NE-AP and LF-AP) toward ELANE and LF, respectively, were evaluated using surface plasmon resonance (SPR). This label-free technique enabled real-time monitoring of the biomolecular interactions. Both aptamers exhibited strong binding to their cognate proteins. The NE-AP/ELANE interaction exhibited high binding affinity with a K_d_ of 31.2 nM (Figures 1E and 1J). Similarly, the LF-AP/LF interaction showed a comparable K_d_ of 28.5 nM. These results confirm the high affinity and specificity of the selected aptamers, thereby establishing a reliable foundation for their application in the sensitive and reproducible detection of ELANE and LF. While, the experimental protocols and corresponding results for the specificity and cross-reactivity evaluations were based on the published work.^32^^,^^33^^,^^34^^,^^35^

### Detection capability of microfiber sensors

To verify the effectiveness of the prepared NE-AP and LF-AP sensors, we tested their transmission spectral response characteristics in 3.57 × 10^−9^ M ELANE solution and 1.28 × 10^−9^ M LF solution,^25^^,^^28^ respectively. Meanwhile, to eliminate interference from external water molecules, testing in double-distilled water (ddH_2_O) was conducted in water so as to preclude any distortion that could be caused during the later spectral normalization process. The experimental results showed that the wavelength shift of the NE-AP sensor in ddH_2_O was 10 nm, whereas the wavelength shift increased significantly to 30.4 nm in 3.57 × 10^−9^ M ELANE solution (Figures 2A and 2B). Similarly, the wavelength shift of the LF-AP sensor in ddH_2_O was 6.6 nm, whereas the wavelength shift of the LF-AP sensor in 1.28 × 10^−9^ M LF solution increased to 25.8 nm (Figures 2C and 2D).

**Figure 2:**
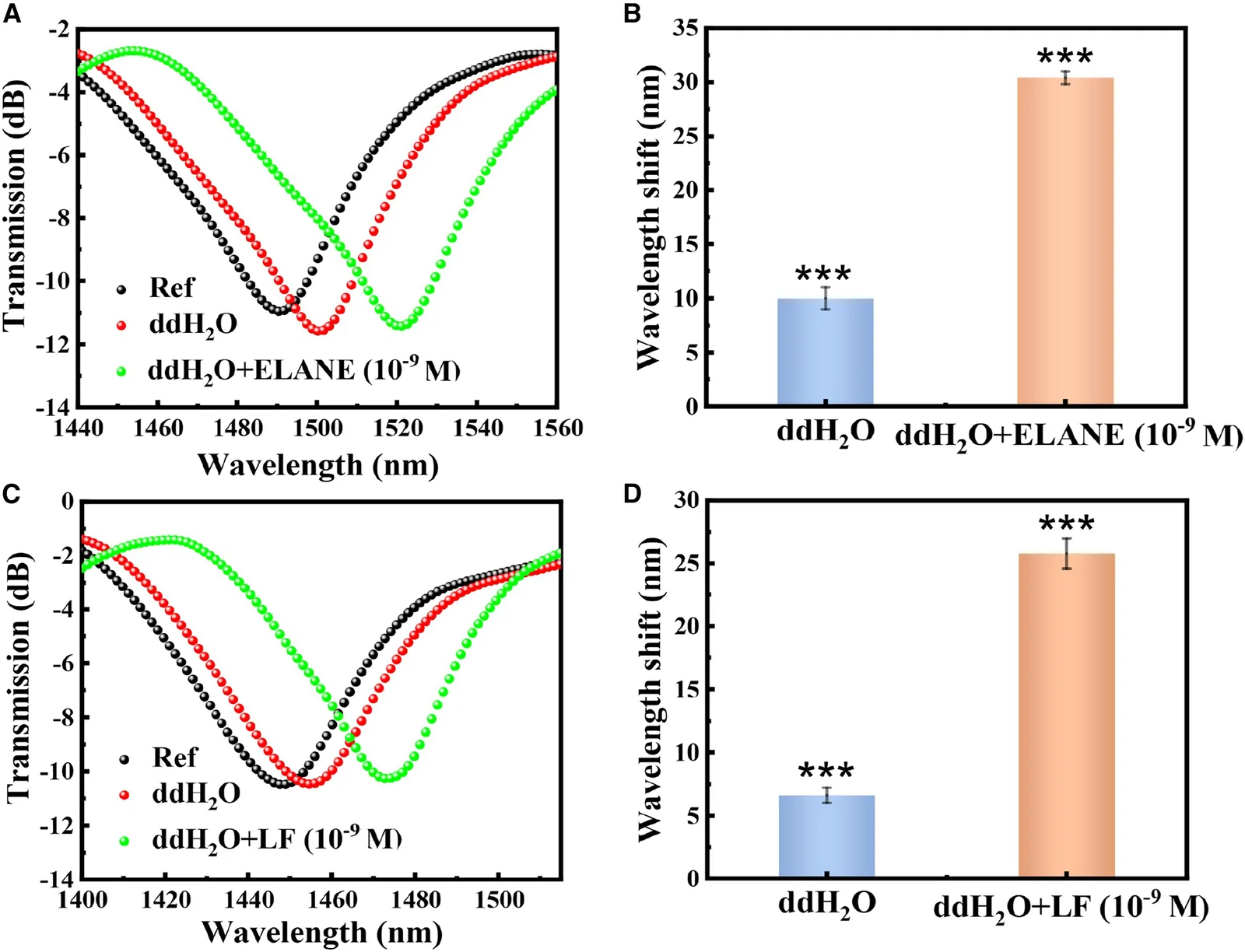
Optical microfiber sensor responses to ELANE and LF proteins (A) Transmission spectra of the NE-AP@GO-modified optical microfiber sensor upon exposure to double-distilled water with or without ELANE protein. (B) Corresponding optical response histogram of the sensor to ELANE protein (derived from A). (C) Transmission spectra of the LF-AP@GO-modified optical microfiber sensor upon exposure to double-distilled water with or without LF protein. (D) Corresponding optical response histogram of the sensor to LF protein (derived from C). Graphs in (B) and (D) represent data derived from average of three measurements of ELANE and LF. Data are presented as mean ± SD. *n* = 6 independently fabricated sensors per concentration point, representing independent sensor devices, with wavelength shifts averaged across the six sensors. ∗∗∗*p* < 0.001 by two-tailed unpaired Student’s *t* test (B and D).

Compared to ddH_2_O, the introduction of target proteins resulted in a notable red shift in the transmission spectral peaks and a significant increase in wavelength shift. This phenomenon confirms the high sensitivity of the NE-AP and LF-AP sensors in detecting ELANE and LF proteins, respectively. The results further validate the effectiveness and reliability of the sensors for target protein detection, providing experimental evidence for their application in the field of biosensing.

### Quantitative detection of the ELANE and LF proteins

In this study, two types of optical microfiber sensors (NE-AP and LF-AP) were systematically evaluated for their detection performance of ELANE and LF proteins, and corresponding control experiments were set up (details can be seen in the figures). The surface morphology of the microfibers before and after the functionalization of the APs/GO interface was observed by scanning electron microscopy (SEM) (Figure 3). Moreover, the Raman spectrograms of the microfiber surface were characterized, confirming successful GO functionalization and aptamer immobilization on the microfiber sensor (Figure S3).

**Figure 3:**
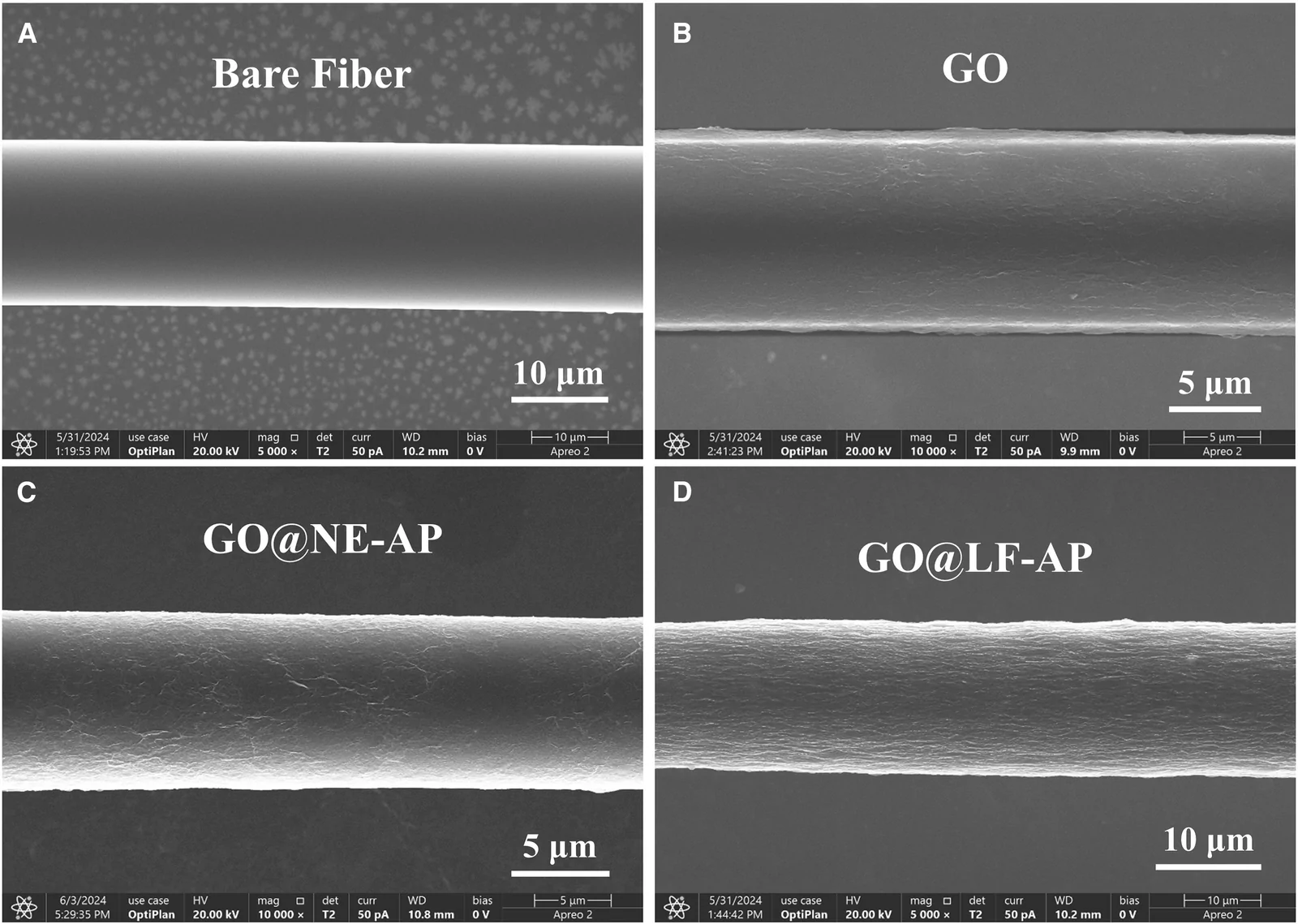
Surface morphology characterization of the optical fibers under different modification conditions (A) SEM image of the bare fiber (unmodified). Scale bars, 10 μm. (B) SEM image of the optical fiber after GO nanointerface functionalization. Scale bars, 5 μm. (C and D) SEM images of the optical fiber after aptamer immobilization (aptamer incubation). Scale bars, 5 μm (C) and 10 μm (D).

The experimental results show that when the NE-AP probe is modified on the surface of the GO-functionalized optical fiber and exposed to an ELANE double-distilled water solution with a concentration range from 3.57 × 10^−20^ M to 3.57 × 10^−12^ M, the sensor exhibits remarkable spectral modulation characteristics. As the concentration of the ELANE solution increases, the spectral transmission peak shows an obvious red-shift phenomenon, with a sensitivity of 1.397 nm/log M and a fitting linearity of 98.02% (Figures 4A and 4B). Notably, the limit of detection (LOD) of this sensor is as low as 3.57 × 10^−20^ M. This excellent detection performance provides important technical support for the trace analysis of ELANE.

**Figure 4:**
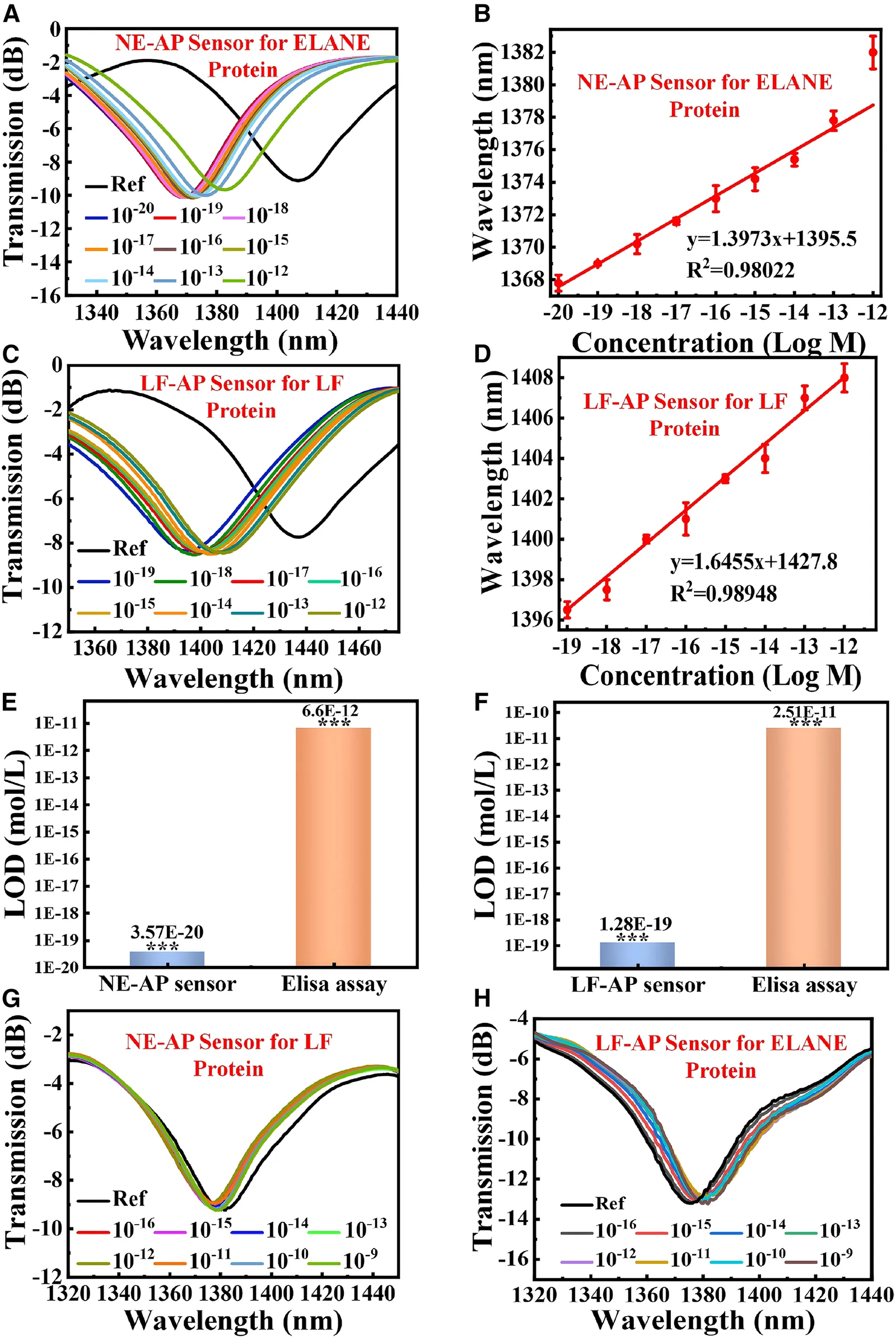
Performance of aptamer@GO-functionalized optical microfiber biosensors for ELANE and LF detection (A) Transmission spectra of the NE-AP@GO-modified optical fiber sensor upon exposure to varying concentrations of ELANE protein. (B) The relationship between wavelength shift and concentration corresponding to (A). (C) Transmission spectra of the LF-AP@GO-modified optical fiber sensor upon exposure to varying concentrations of LF protein. (D) The relationship between wavelength shift and concentration corresponding to (C). (E and F) Comparison of limits of detection between the NE-AP biosensor and traditional ELISA for ELANE, as well as between the LF-AP biosensor and traditional ELISA for LF. (G) Spectral response of the NE-AP@GO microfiber sensor to LF protein with concentrations ranging from 3.57 × 10^−16^ M to 3.57 × 10^−9^ M for cross-reactivity test. (H) Spectral response of the LF-AP@GO microfiber sensor to ELANE protein with concentrations ranging from 1.28 × 10^−16^ M to 1.28 × 10^−9^ M for cross-reactivity test. Graphs in (E)–(F) represent data derived from average of three measurements of ELANE-AP and LF-AP. Data are presented as mean ± SD. *n* = 6 independently fabricated sensors per concentration point, representing independent sensor devices, with wavelength shifts averaged across the six sensors. *n* = 10 consecutive measurements of ddH_2_O on each of six independent sensors. ∗∗∗*p* < 0.001 by two-tailed unpaired Student’s *t* test (E and F).

Similarly, when the LF-AP probe is modified on the surface of the GO-functionalized optical fiber and exposed to an LF double-distilled water solution with a concentration range from 1.28 × 10^−19^ M to 1.28 × 10^−12^ M, a red-shift of the spectral transmission peak is also observed. Its sensitivity is 1.65 nm/log M, the fitting linearity is 98.95%, and the LOD for LF is as low as 1.28 × 10^−19^ M (Figures 4C and 4D).

The LOD was determined using the 3σ/slope method (IUPAC). The blank standard deviation was obtained from 10 consecutive measurements of ddH_2_O on six independent sensors. The calibration slope was derived from the linear regression of wavelength shift vs. log(concentration) in the linear dynamic range. Specifically, for the NE-AP sensor, *σ*_blank_ = 0.036 nm and *S* = 1.397 nm/log M, yielding an LOD of 3.57 × 10^−20^ M. For the LF-AP sensor, *σ*_blank_ = 0.048 nm and *S* = 1.65 nm/log M, yielding an LOD of 1.28 × 10^−19^ M (Methods S2).

The apparent discrepancy between the aptamer’s solution-phase dissociation constant (Kd ≈ 30 nM) and the sensor’s bulk LOD (10^−20^ M) could be rationalized by the high preconcentration capacity of the GO nanointerface. GO possesses an exceptionally large specific surface area and abundant oxygen-containing groups that facilitate the adsorption and local enrichment of proteins at the sensing interface. Based on the Langmuir adsorption model, the partition coefficient (Kp) of serum proteins on GO has been reported to be on the order of 10^6^–10^8^,^24^^,^^36^ implying that the local concentration of ELANE/LF at the GO surface can reach the nM range even when the bulk concentration is as low as 10^−20^ M. This local enrichment effect effectively overcomes the bulk-phase diffusion limitation and allows the sensor to exploit the full binding capacity of the immobilized aptamers.

The LODs of ELANE and LF proteins obtained using the NE-AP and LF-AP biosensors were compared with those achieved by the conventional ELISA method (Figures 4E and 4F). The aptamer-based sensors exhibited remarkably lower LODs for the target proteins—approximately eight orders of magnitude lower than those of the ELISA assay—indicating the superior sensitivity of the proposed biosensing platforms.

Additionally, Conventional ELISA detection typically requires several hours to 45 min to complete.^37^^,^^38^ In contrast, the platform-integrated microfluidic sample processing and real-time optical detection technology reduced the measurement step (from sample injection to signal acquisition) to approximately 3 min—significantly shorter than traditional ELISA (which requires several hours to 72 h to complete). The total turnaround time, including sample dilution and sensor equilibration, was approximately 15–20 min, which remained substantially faster than conventional methods. The performance comparison of sensor fabricated with the conventional methods could be found in Table S1.

The remarkably low LOD can be attributed to the synergistic effect between GO and the immobilized aptamers. GO possesses a two-dimensional sheet-like structure with atomic-layer thickness and an exceptionally large specific surface area, which offers an ideal substrate for the high-density and ordered immobilization of aptamers. This facilitates the capture of a greater number of target proteins per unit area and amplifies the corresponding detection signal. To experimentally validate this contribution, we compared the sensitivity of three types of sensors: bare microfiber, GO-coated microfiber without aptamer (passivated with BSA), and GO-aptamer functionalized sensor. At the same ELANE concentration (3.57 × 10^−9^ M), the GO-aptamer sensor exhibited a wavelength shift approximately 6-fold greater than that of the bare fiber and 4-fold greater than that of the GO-only sensor, confirming that aptamer-specific binding on the GO scaffold is the primary driver of the enhanced signal.

### Selectivity of sensor

In practical detection, fluctuations of interfering molecules in the extracellular environment are inevitable and thus must be carefully considered and discussed when establishing detection conditions.^25^ To verify whether the sensors respond exclusively to specific target proteins, we tested the specificity of both sensors using non-target proteins. Specifically, the NE-AP sensor was used to detect LF protein at concentrations ranging from 1.28 × 10^−16^ M to 1.28 × 10^−9^ M. The results showed that the wavelength shift of the NE-AP sensor in response to LF protein was almost negligible compared to the original transmission spectrum (Figure 4G), indicating that LF protein did not bind to the NE aptamer modified on the sensor surface, thereby demonstrating the high specificity of the sensor. Similarly, the LF-AP sensor was used to detect ELANE protein at concentrations ranging from 3.57 × 10^−16^ M to 3.57 × 10^−9^ M, and no significant changes were observed in the transmission spectrum (Figure 4H). These experimental results further validate the reliability of the sensors, confirming that the detection signals originate solely from the specific binding between the target proteins and their corresponding aptamers, rather than environmental noise or other non-specific interference factors.

Beyond aptamer cross-reactivity, non-specific adsorption (NSA) of abundant serum proteins represents a distinct challenge for biosensing in complex biological matrices. Although dedicated BSA/HSA challenge experiments were not performed in this study, two lines of evidence from our current data indirectly support the sensor’s resistance to NSA. First, the scrambled aptamer control experiments described that *in vivo* evaluation of the sensor in a murine lipopolysaccharide (LPS)-induced inflammation model produced no significant spectral shifts when exposed to serum samples, confirming that non-aptamer-mediated protein adsorption to the GO or fiber surface does not generate a measurable signal. Second, healthy serum samples induced minimal spectral shifts (Δ*λ* < 5 nm), whereas septic serum produced pronounced shifts (Δ*λ* > 30 nm for ELANE), despite both sample types containing similar high concentrations of abundant proteins such as albumin and immunoglobulins. This stark contrast strongly suggests that the observed signals are driven by elevated ELANE and LF concentrations rather than by non-specific adsorption. Nevertheless, the systematic quantitative evaluation of NSA using high-concentration BSA/HSA challenge and serum spike-recovery experiments provide an important direction for future assay optimization.

### *In vivo* evaluation of the sensor in a murine LPS-induced inflammation model

To evaluate the *in vivo* performance of GO-functionalized microfiber sensors, we established a LPS-induced systemic inflammation model in mice (*n* = 3 per group) to simulate neutrophil activation in sepsis and chronic inflammatory states. The experimental mice received intraperitoneal LPS injections, with serum samples collected at 1, 24, and 96 h post-injection for analysis using NE-AP and LF-AP sensors (Figure 5A). Additionally, prior to analysis, all samples were diluted 1:10 in phosphate-buffered saline (PBS, pH 7.4). Each diluted sample was then introduced into the sensor chamber at a constant flow rate of 10 μL/min via a syringe pump. The sensor response was monitored in real-time.

**Figure 5:**
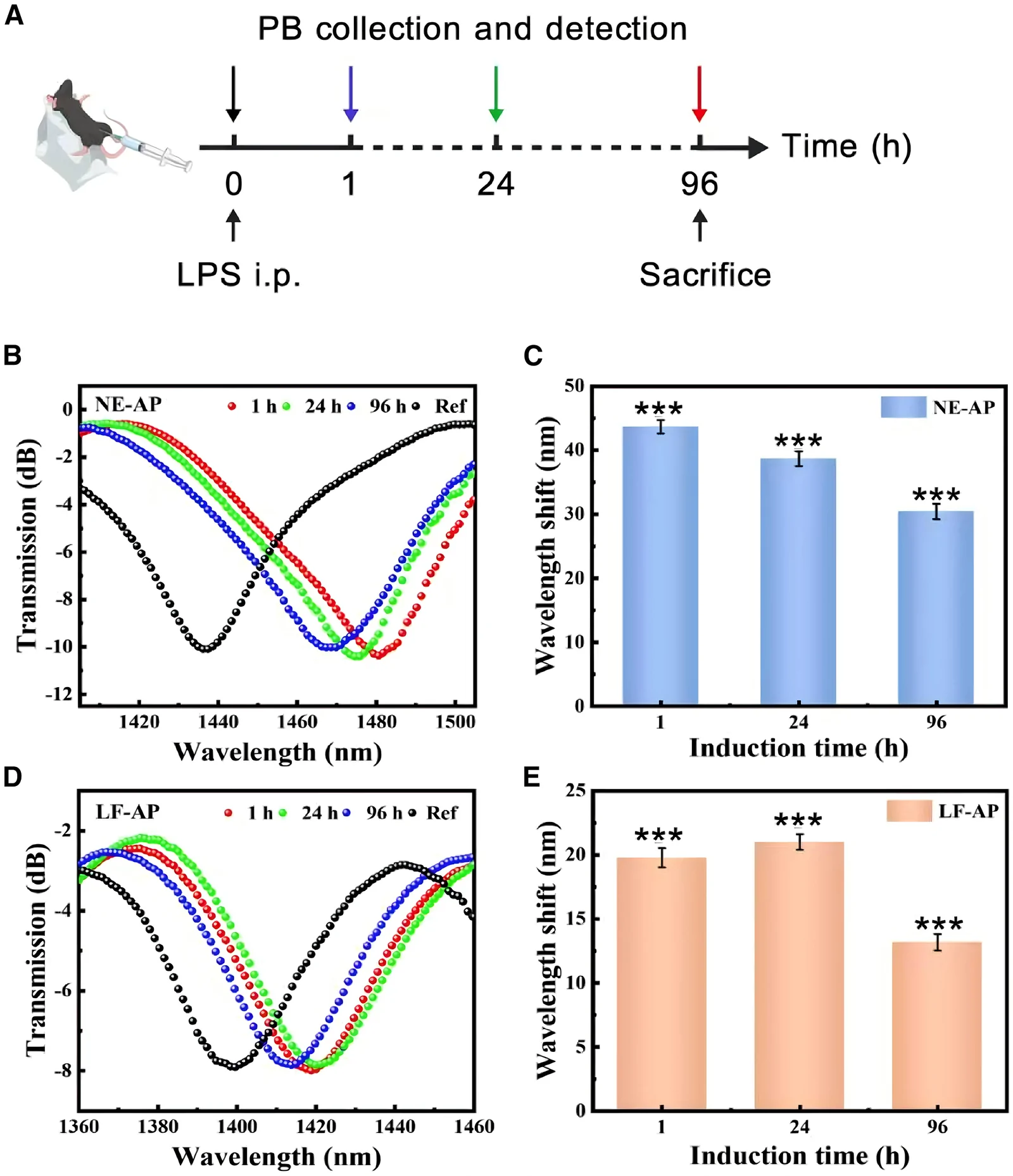
Spectral analysis of serum biomarkers in LPS-induced inflammatory mice using functionalized microfiber sensors (A) Serum samples collected from mice at 1, 24, and 96 h post-injection. (B) Transmission spectra of serum samples from mice #1–3 at different time points (1, 24, and 96 h) post-LPS injection detected by NE-AP@GO sensor. (C) Corresponding wavelength shifts and temporal trends for (B). (D) Transmission spectra of serum samples from mice #4–6 detected by LF-AP@GO sensor. (E) Corresponding wavelength shifts and temporal trends for (D). Data are presented as mean ± SD. *n* = 6 independently fabricated sensors for different time. ∗*p* < 0.05, ∗∗∗*p* < 0.001 by two-tailed unpaired Student’s *t* test (C and E).

Meanwhile, to evaluate the reusability of the aptamer-functionalized sensors, regeneration cycles were performed using 10 mM NaOH (pH 12.0) to dissociate bound analytes.^25^^,^^28^^,^^29^ The sensors retained more than 92% of their initial response after 15 regeneration cycles, with signal degradation remaining below 8%. After 20 cycles, the response decreased to approximately 85% of the initial value, likely due to gradual aptamer denaturation or loss from the GO surface. These results confirm that the sensor platform offers satisfactory reusability for practical applications, with 10–15 cycles being the recommended operational limit to ensure measurement reliability.

Following each measurement, a regeneration step was performed by flushing the chamber with 10 mM NaOH (pH 12.0) to dissociate bound analytes and regenerate the aptamer-functionalized surface for the next cycle. In parallel, control experiments were performed. First, PBS alone was injected to establish a stable baseline. Second, samples were tested on a sensor functionalized with a scrambled nucleic acid sequence to assess the extent of non-specific binding.

The sensors exhibited significant spectral modulation responses. One hour after LPS injection, the NE-AP sensor detected a significant redshift (Δ*λ* = 43.8 nm) in the transmission spectrum (Figures 5B and 5C), indicating rapid neutrophil activation and the release of ELANE. This response attenuated to Δ*λ* = 38.8 nm at 24 h and further declined to Δ*λ* = 30.4 nm by 96 h, consistent with the resolution pattern of acute inflammation. Similarly, the LF-AP sensor demonstrated elevated LF levels, peaking at 24 h (Δ*λ* = 21 nm) and decreasing to Δ*λ* = 13.2 nm at 96 h (Figures 5D and 5E), correlating well with the temporal decline in neutrophil activity. Moreover, it could be seen that the response spectrum denominated a redshift phenomenon, proving that the concentrations of ELANE and LF proteins in serum were higher than 10^−9^ M (Figure 5).

These findings demonstrate the sensor’s capability for real-time monitoring of neutrophil activation dynamics through ELANE and LF quantification. The distinct temporal patterns—immediate ELANE release versus delayed LF response—enable discrimination between acute and chronic inflammatory phases. While these *in vivo* results confirm the sensor’s feasibility in an animal model, further clinical studies with human samples are warranted to assess its diagnostic utility in complex biological environments.

### Clinical sepsis serum analysis

While the preceding section established the sensor’s feasibility in a murine model, we next performed a preliminary validation using human serum samples to explore its potential clinical utility. This study investigates the clinical detection of sepsis using a custom-developed optical fiber sensor for serum analysis. Sepsis, a life-threatening systemic inflammatory response syndrome triggered by infection, requires early and accurate diagnosis to reduce mortality.^39^^,^^40^ The optical fiber probe was functionalized with nucleic acid aptamers specifically targeting ELANE and LF, two key biomarkers associated with sepsis, to achieve highly specific capture of these molecules. Serum samples for detection were collected from patients diagnosed with sepsis and from healthy volunteers. The clinical and inflammatory characteristics of the sepsis and control groups are summarized in Table 1.

**Table 1:** Comparison of clinical and inflammatory profiles between septic patients and healthy controls

Category	CTRL	Sepsis
Number	11	8
Age	57.6 ± 14.4	69.4 ± 12.0
Male	9	4
Female	2	4
CRP mg/L	28.3 ± 25.4	135.6 ± 61.9
WBC (mean ± SD)	12.8 ± 3.7	17.8 ± 26.7
IL-6 (mean ± SD)	0.041 ± 0.026	72.2 ± 25.0
PCT (mean ± SD)	17.1 ± 9.6	406.6 ± 47.0

The diagnostic value was obtained by combining known ELANE or LF concentrations with clinical diagnosis (Figure 6). However, due to the limited sample size (*n* = 8 sepsis patients, *n* = 11 healthy controls), we did not perform receiver operating characteristic (ROC) curve analysis or derive diagnostic cutoff values, as such analyses would be statistically underpowered and potentially misleading. Instead, we present the spectral responses of the NE-AP and LF-AP sensors to serum samples from both groups as descriptive comparisons. These preliminary data serve as a proof-of-concept demonstration that the sensor can differentiate between septic and healthy sera under controlled conditions, but formal diagnostic performance metrics (sensitivity, specificity, and area under the curve [AUC]) await validation in larger, independent cohorts.

**Figure 6:**
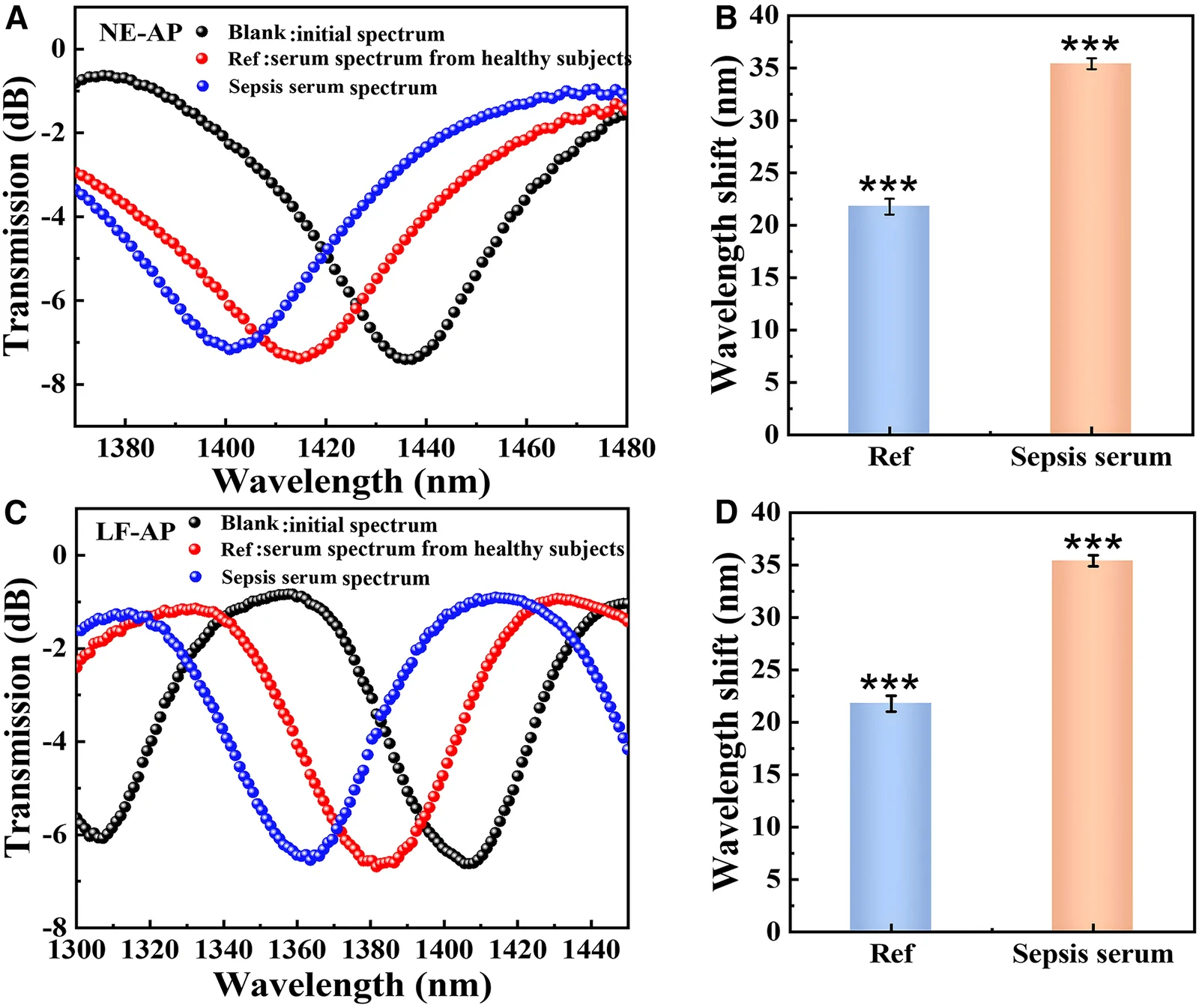
Clinical detection of serum samples from healthy and septic patients with aptamer@GO microfiber sensors (A–D) Transmission spectra of serum samples from healthy controls and septic patients, detected with the NE-AP@GO sensor (A) and the LF-AP@GO sensor (C), respectively. (B and D) Wavelength shifts of serum samples from healthy controls and septic patients obtained with (A) NE-AP and (C) LF-AP sensors. Data are presented as mean ± SD from serum of healthy subject and patients with sepsis. ∗∗∗*p* < 0.001 by two-tailed unpaired Student’s *t* test (B and D).

The transmission spectra obtained from the NE-AP and LF-AP sensors revealed distinct differences between sepsis patients and healthy controls in this preliminary cohort (Figure 6). Serum from healthy individuals induced minimal spectral shift with stable signal profiles, whereas samples from septic patients produced pronounced spectral shifts (NE-AP: Δλ = 42.6 ± 12.8 nm for sepsis vs. 4.2 ± 2.1 nm for healthy controls; LF-AP: Δλ = 31.5 ± 9.7 nm vs. 3.8 ± 1.9 nm). These results suggest that the observed optical changes are likely due to elevated concentrations of ELANE and LF in septic serum, which alter the optical properties of the sensor interface. While these findings are encouraging, we emphasize that they are preliminary and require validation in larger, well-powered clinical studies to establish definitive diagnostic performance.

## Discussion

In this study, we developed a high-sensitivity and high-specificity optical microfiber sensor modified with GO for rapid detection of neutrophil activation by targeting two key biomarkers: ELANE and LF. The sensor utilizes GO’s high surface area and biocompatibility to immobilize DNA aptamers that specifically bind to ELANE and LF, enhancing target protein-binding efficiency. Detection is based on changes in the surface refractive index of the optical microfiber upon aptamer-protein binding, resulting in spectral shifts that enable real-time quantitative analysis with extremely low LODs (3.57 × 10^−20^ M for ELANE and 1.28 × 10^−19^ M for LF). The sensor demonstrates excellent selectivity, and the use of aptamers as recognition elements avoids the cross-reactivity issues associated with antibodies.

The mechanistic basis for the exceptional sensitivity can be systematically summarized as a triple amplification strategy integrating chemical, optical, and physical enhancements:

(i) Chemical amplification (GO scaffold): the two-dimensional GO nanosheet provides an ultra-high specific surface area and abundant oxygen-containing functional groups, enabling high-density aptamer immobilization via π-π stacking and electrostatic interactions. This ordered, upright orientation of aptamers promotes multivalent interactions with target proteins, amplifying the effective refractive index change per bound protein molecule.
(ii) Optical amplification (evanescent field enhancement): the GO nanosheet possesses a high refractive index (∼2.1) compared to water (∼1.33), which significantly enhances the local evanescent field intensity at the microfiber waist region. This increases the sensor’s sensitivity to refractive index changes induced by surface binding events.
(iii) Physical amplification (flow-enhanced mass transport): the integration of the sensor with a microfluidic flow cell (constant flow rate of 10 μL/min) overcomes diffusion-limited mass transport at sub-picomolar concentrations, enabling efficient analyte capture even at attomolar levels.

This triple amplification strategy—chemical, optical, and physical—working in concert underpins the attomolar-level LODs achieved in this work.

Despite promising results, several limitations require further optimization. The ultra-thin waist of the tapered fiber makes it fragile and limits the upper detection range due to rapid aptamer saturation. Integrating the sensor with microfluidic chips to allow continuous sample flow could mitigate this issue.^41^ The manufacturing process of tapered fibers is also delicate, requiring precise control of parameters such as heating and stretching, which may affect sensor reproducibility. Standardizing the manufacturing process and optimizing parameters could enhance sensor repeatability and reliability. Furthermore, while the sensor performs well in controlled laboratory settings, its performance in real clinical environments needs to be validated through clinical trials involving patient samples.

Nevertheless, the GO-modified optical microfiber sensor represents a significant advancement in neutrophil activation detection. Its high sensitivity, real-time detection capability, and miniaturization potential make it a promising tool for early diagnosis and monitoring of sepsis and chronic inflammation.

The preliminary clinical validation with 8 septic patients and 11 healthy controls demonstrated promising signal differentiation between the two groups. However, we acknowledge that this sample size is insufficient to derive statistically robust diagnostic performance metrics such as ROC curves, AUC values, sensitivity, or specificity. Moreover, the current cohort did not include patients with non-septic inflammatory conditions (e.g., trauma, surgery, and autoimmune flare), which would be essential to assess the true clinical specificity of the sensor. Therefore, our clinical data should be interpreted as a feasibility demonstration rather than a validated diagnostic assay. Future work will focus on (1) expanding the clinical cohort to include diverse patient populations with varying inflammatory etiologies, (2) systematically establishing diagnostic cutoff values through receiver operating characteristic analysis, and (3) validating the performance of the sensor in prospective, multicenter studies.

In summary, we developed a GO-aptamer-modified microfiber sensor, where transmission peak shifts result from protein-aptamer binding-induced refractive index changes. It was shown that the sensor is capable of detecting ELANE and LF in human blood with high sensitivity and specificity and that the NE-AP sensor is capable of directly determining ELANE levels between 3.57 × 10^−20^ M and 3.57 × 10^−12^ M and exhibits a good linear response to ELANE concentrations (R^2^ = 0.9802). The corresponding LOD was determined to be 3.57 × 10^−20^ M. The LF-AP sensor was capable of directly determining LF levels between 1.28 × 10^−19^ M and 1.28 × 10^−12^ M, showing a good linear response to LF concentrations (R^2^ = 0.9895). The corresponding limit of detection was determined to be 1.28 × 10^−19^ M. The developed sensor demonstrated a significantly lower LOD compared to conventional ELISA. The feasibility of the developed optical fiber sensor was preliminarily verified in a murine model and further validated with clinical serum samples from septic patients. It provides a sensitive research tool for studying neutrophil degranulation dynamics and shows preliminary promise for sepsis-related applications. However, its clinical utility remains to be established through rigorous validation in large, well-designed prospective cohorts, which will be the focus of our ongoing work.

### Limitations of the study

Several limitations of the current study warrant acknowledgment. First, while our control experiments with scrambled aptamers and healthy serum indirectly suggest minimal interference from non-specific adsorption, a more rigorous quantitative characterization of NSA—including high-concentration BSA/HSA challenge and spike-recovery experiments in diluted serum—would further strengthen the evidence for the sensor’s robustness in complex clinical matrices. Future studies incorporating surface blocking strategies (PEGylation or BSA pre-coating) may further minimize any residual NSA. Second, the ultra-thin waist of the tapered fiber makes it fragile and limits the upper detection range due to rapid aptamer saturation. Integrating the sensor with microfluidic chips to allow continuous sample flow could mitigate this issue. Third, the manufacturing process of tapered fibers is delicate, requiring precise control of parameters such as heating and stretching, which may affect sensor reproducibility. Standardizing the manufacturing process and optimizing parameters could enhance sensor repeatability and reliability. Fourth, while the sensor performs well in controlled laboratory settings, the clinical validation cohort in this study was limited in size (*n* = 8 sepsis patients, *n* = 11 healthy controls) and did not include patients with other inflammatory conditions that could serve as important disease controls. Consequently, we are unable to provide definitive diagnostic performance metrics such as ROC curves, AUC values, sensitivity, or specificity. The clinical data presented here should therefore be considered preliminary proof-of-concept results. Large-scale, prospective studies with diverse patient populations are essential to establish the sensor’s true diagnostic utility and to determine optimal cutoff values for clinical decision-making.

## Resource availability

### Lead contact

Requests for further information and resources should be directed to and will be fulfilled by the lead contact, Jijun Feng (fjijun@usst.edu.cn).

### Materials availability

This study did not generate new unique reagents or materials.

### Data and code availability

• This paper does not report original code.
• All data associated with this study are present in the article or the supplemental information. All data reported in this article will be shared by the lead contact upon request.
• Any additional information required to reanalyze the data reported in this article is available from the lead contact upon request.

## Acknowledgments

The work was supported by the 10.13039/501100001809National Natural Science Foundation of China (U23A20381), 10.13039/501100003399Science and Technology Commission of Shanghai Municipality (23010503600 and 23530730500), Natural Science Foundation of Shanghai (24ZR1441300), and Shanghai Oriental Talents Program (2023DFYCJH). Additionally, authors are grateful to Dr. Pu Zou for the helpful and enlightening discussions.

## Author contributions

Z.Y., conceptualization, methodology, investigation, validation, and writing – original draft; H.D., conceptualization, methodology, investigation, validation, and writing – original draft; X.C., conceptualization, methodology, and investigation; M.X., investigation, validation, and formal analysis; R.L., conceptualization, methodology, investigation, validation, and formal analysis; J.J.W., conceptualization, investigation, and review and editing; C.Y., conceptualization, supervision, and writing and editing; K.W., conceptualization and methodology; X.Z., methodology, investigation, and validation; H.J., conceptualization, methodology, investigation, supervision, review and editing, and funding acquisition; J.F., conceptualization, supervision, writing – review and editing, project administration, and funding acquisition.

## Declaration of interests

The authors declare no competing interests.

## STAR★Methods

### Key resources table

**Table:** 

REAGENT or RESOURCE	SOURCE	IDENTIFIER
**Biological samples**		

Serum samples from healthy individuals for quality control (QC) (Age: 57.6 ± 14.4, Sex: 9 males and 2 females)	Renji Hospital, School of Medicine, Shanghai Jiao Tong University	Ethics Approval No. KY2021-060-A
Serum samples from Sepsis individuals (Age: 69.4 ± 12.0, Sex: 4 males and 4 females)	Renji Hospital, School of Medicine, Shanghai Jiao Tong University	Ethics Approval No. KY2021-060-A
Serum samples from healthy mice for quality control (QC)	Shanghai Jiao Tong University School of Medicine	Ethics Approval No. KY2021-060-A
Serum samples from Sepsis mice induced by lipopolysaccharides	Shanghai Jiao Tong University School of Medicine	Ethics Approval No. KY2021-060-A

**Chemicals, peptides, and recombinant proteins**		

Concentrated sulfuric acid (H_2_SO_4_)	Sigma-Aldrich	CAS#: 7664–93-9
Hydrogen peroxide (H_2_O_2_)	Sigma-Aldrich	CAS#: 7722–84-1
3-aminopropyltriethoxysilane (APTES)	Sigma-Aldrich	CAS#: 919–30-2
Ethanol (C_2_H_6_O)	Sigma-Aldrich	CAS#: 64–17-5
Graphene oxide (GO)	Sigma-Aldrich	CAS#: 7782–42-5
Double distilled water (ddH_2_O)	N/A	N/A
Neutrophil elastase (ELANE)	Wuhan Fine Biotech Co., Ltd.	CAS#: 39445–21-1
Lactoferrin (LF)	Sigma-Aldrich	CAS#: 112163–33-4
SDS-PAGE sample buffer (solution)	Sigma-Aldrich	N/A
SDS	Sigma-Aldrich	CAS#: 151–21-3
Tris-HCl	Sigma-Aldrich	CAS#: 1185–53-1
Bromophenol blue	Sigma-Aldrich	CAS#: 115–39-9
Thomas Brilliant Blue	Sigma-Aldrich	CAS#: 3844–45-9
SPR running buffer (solution)	Sigma-Aldrich	N/A
N-hydroxysuccinimide	Sigma-Aldrich	CAS#: 6066–82-6
N-ethyl-N′(dimethylaminopropyl) carbodiimide	Sigma-Aldrich	CAS#: 25952–53-8
Ethanolamine hydrochloride	Sigma-Aldrich	CAS#: 2002–24-6
10 mM NaOH solution	Sinopharm Chemical Reagent Co., Ltd.	CAS#: 1310–73-2
Lipopolysaccharides	Sigma-Aldrich	CAS#: 93572–42-0
Poly (butylene succinate)	Sigma-Aldrich	CAS#: 25777–14-4

**Experimental models: Organisms/strains**		

BALB/c nude mice (3–4 weeks old)	Beijing Huafukang Bioscience Technology Co., Ltd.	(RRID: IMSR: BALB/c-nude)
BALB/c nude sepsis mice (3–4 weeks old) induced by LPS	Beijing Huafukang Bioscience Technology Co., Ltd.	(RRID: IMSR: BALB/c-nude with sepsis)

**Oligonucleotides**		

NE-AP aptamer	This paper	CACGATGGTTAGGCGGGC CTTGAGGCTAATAATGTTGTTA
LF-AP aptamer	This paper	AGGCAGGACACCGTAACCG GTGCATCTATGGCTACTAGCT TTTCCTGCCT

**Software and algorithms**		

Origin 2020	OriginLab Corporation	https://www.originlab.com/2020C
BioGDP	Guangzhou Ruijing Information Technology Co., Ltd.	https://biogdp.com/zh
GraphPad Prism version 10.0	GraphPad Software	https://www.graphpad.com/
ImageJ	USA National Institutes of Health	https://www.imagej.com.cn/
BIA Evaluation Software (version 3.2)	Biacore AB	N/A
NUPACK	California Institute of Technology	https://www.nupack.org/
RNAComposer	MPM BioImpact	https://rnacomposer.cs.put.poznan.pl/
PDBePISA	Protein DataBank in Europe	https://www.ebi.ac.uk/msd-srv/prot_int/pistart.html
HDOCK server	Huazhong University of Science and Technology	https://hdock.phys.hust.edu.cn
AlphaFold Protein Structure Database	Google DeepMind and	EMBL-EBI
PyMOL	Schrödinger	https://www.pymol.org/

**Other**		

Single-mode optical fiber (SMF-28, with a cladding diameter of 125 μm)	Corning Incorporated	N/A
BIAcore T200 biosensor system	Cytiva	N/A
AQ6370E spectral analyzer	YOKOGAWA	N/A
SC-5 supercontinuum spectral light source	Wuhan Anyang Laser Technology Co., Ltd.	N/A

### Experimental model and study participant details

#### Human sample preparation

Serum samples used for detection were from sepsis patients and healthy volunteers. A total of 19 participants were enrolled in this study, comprising 11 healthy controls (HCs) and 8 patients diagnosed with sepsis, additionally, the age and sex were provided in Table 1. All participants were recruited from Renji Hospital, School of Medicine, Shanghai Jiao Tong University, between January 2025 and June 2025. Patients diagnosed with sepsis were recruited from the Department of Critical Care Medicine, and the diagnosis of sepsis was confirmed by attending physicians based on the Sepsis-3 criteria (SOFA score ≥2 and suspected infection). During the same period, healthy volunteer controls who underwent routine physical examinations at our hospital were recruited. These individuals had no chronic illnesses, history of medications, or abnormalities on routine blood tests. The exclusion criteria were (1) age <18 years; (2) pregnancy or lactation; (3) history of immunosuppressive or antibiotic therapy within 2 weeks; (4) patients with a condition complicated by other diseases (e.g., malignancy, autoimmune disorders, or chronic inflammatory diseases); (5) unwillingness to participate in the study or uncooperative with the researchers; and (6) unavailability of complete clinical data. All participants are of Han Chinese ethnicity and provided written informed consent, and all human studies were conducted in accordance with the Declaration of Helsinki. Peripheral venous blood samples were collected from the patients with sepsis at the time of diagnosis, before the initiation of any therapeutic intervention. All participants donated their blood samples in the early morning after fasting overnight for over 8 h. Blood samples were collected into sterile tubes without anticoagulant and allowed to clot at room temperature for 30 min. After clotting, Samples were centrifuged at 1500×g for 15 min at 4°C, and the resulting serum was separated and stored at −20°C for subsequent analysis. This study and the animal experiments were conducted in accordance with the Declaration of Helsinki and approved by the Institutional Review Board (or Ethics Committee) of Shanghai Jiao Tong University School of Medicine, Renji Hospital (Approval No. KY2021-060-A). Additionally, no cell lines were used in this study.

#### Sepsis mouse model

The animals used in this study were all male mice (three-to four-week-old), weighing approximately 15–20 g. The mice were from the Animal Facility of Shanghai Jiao Tong University School of Medicine] and held under specific pathogen-free (SPF) conditions. The animals were housed in a temperature- (22 ± 2°C) and humidity-controlled (50 ± 10%) vivarium with lights maintained on a 14:10 light/dark cycle, with *ad libitum* access to standard chow and filtered water. Systemic inflammation was induced by intraperitoneal injection of lipopolysaccharide (LPS) at a dose of 10 mg/kg. Peripheral blood samples were collected from the orbit at 1 h, 24 h, and 96 h post-injection. Blood samples were placed in non-anticoagulant tubes and tilted at room temperature for 45 min to promote complete clotting, followed by centrifugation at 1500×g for 15 min at 4°C. Serum samples were obtained and stored at −20°C for subsequent analysis. After the experiments, the mice were euthanized.

### Method details

#### SDS-page analysis

After centrifugation of the protein samples at 15000 g for 5 min, the precipitates were collected and dissolved in a specific sample buffer. This buffer contained 10 mM Tris-HCl (pH 6.8), 10% glycerol, 0.05% bromophenol blue, 2% Sodium Lauryl Sulfate (SDS), and 5% 2-mercaptoethanol. Subsequently, the solubilized protein samples were subjected to SDS-PAGE analysis along with protein molecular weight markers of known molecular mass. The electrophoresis process was carried out strictly following the experimental method reported by Jia et al.^28^ At the end of electrophoresis, the gel was stained using Thomas Brilliant Blue to visualize the protein bands.^25^

#### SPR assay

To accurately determine the binding affinity of specific aptamers, we used a BIAcore T200 biosensor system for surface plasmon resonance (SPR) analysis. The experimental methodology was described in detail according to Liu et al.^25^ During the experiment, the carboxymethylated sensor chip (Cytiva) was first pre-equilibrated using running buffer (10 mM HEPES, pH 7.5, 150 mM NaCl, 1 mM MgCl_2_, 1 mM CaCl_2_, 2.7 mM KCl). Subsequently, the chip surface was activated by a mixture of 100 mM N-hydroxysuccinimide and 400 mM N-ethyl-N′(dimethylaminopropyl) carbodiimide in order to immobilize the His-tagged ELANE and LF proteins onto it. After protein immobilization, the chip surface was deactivated using 1 M ethanolamine hydrochloride (pH 8.5) to block any residual activation groups.

After ensuring baseline stabilization, we introduced samples of at least six different concentrations of NE-AP and LF-AP aptamers into flow cells. After each sample injection, the protein surface was regenerated using a 10 mM NaOH solution and the chip was re-equilibrated with running buffer. Finally, SPR sensor profiles were analyzed in depth using BIA Evaluation Software (version 3.2, BIAcore) to accurately determine the binding affinity between the aptamer and the target protein.^25^

#### Molecular docking

The LF-AP and NE-AP aptamers were prepared following the previously published works, where LF-AP was originally screened against target YFL-1 using Microarray SELEX,^32^ and NE-AP was derived from the NE inhibitor DD18 using Splint SELEX.^33^ Notably, due to the multiple aptamers identified in the published work, the YFL-1 and DD18 were selected as candidate aptamers in this work. Meanwhile, based on the sequences reported,^32^^,^^33^^,^^34^ the YFL-1 and DD18 aptamers were obtained through gene synthesis, designated LF-AP and NE-AP aptamers, respectively (Methods S1). The secondary structure prediction of NE-AP and LF-AP was completed by NUPACK. The tertiary structure of aptamer was predicted from RNAComposer, and the protein structure was downloaded from AlphaFold Protein Structure Database. Then, perform protein-DNA docking using the HDOCK server, analyze interaction results with PDBePISA, and visualize findings with PyMOL.^35^

#### Fabrication and GO-functionalized sensors

Tapered microfibers with waist diameters of 9 μm and 9.6 μm were fabricated using the fused taper method. The fibers were locally heated and simultaneously stretched at both ends in a molten state.^25^^,^^36^ To achieve high reproducibility of the tapered microfibers prepared, a programmed fusion splicer-based tapering process was adopted. The single-mode fiber (SMF) was secured in the clamp fixed, with its distal end connected to the broadband light source (BLS) and the optical spectrum analyzer (OSA), respectively. Additionally, the flow rates of hydrogen and oxygen were set to 300 mL/min and 600 mL/min, respectively. The fiber-optic platforms moved toward each other at a speed of 50 μm/s, and the tapering process was maintained for 5 min and terminated once the target spectrum was observed on the optical spectrometer, thereby a tapered fiber was obtained (Figure S1). When light propagates through the fiber, total internal reflection occurs at the core-cladding interface. As the light passes through the tapered waist region, the evanescent field outside the core is enhanced due to the gradual reduction in diameter.^24^ The adsorption of biomolecules onto the fiber surface alters the refractive index of the surrounding medium, resulting in a wavelength shift of the transmitted light. By monitoring this wavelength shift, refractive index detection can be achieved.^28^

Prior to functionalization, the fibers were cleaned with piranha solution to remove surface contaminants, followed by thorough rinsing with deionized water. The fiber surface was then functionalized with 3-APTES silane coupling agent, whose hydrolyzed groups formed covalent bonds with surface hydroxyl groups, while exposing amino groups for subsequent modification.^25^^,^^37^ Finally, graphene oxide solution prepared by GO powder and ddH_2_O with the mass of 1:1, was coated onto the modified microfiber surface through electrostatic interactions between the oxygen-containing groups on graphene oxide and the amino groups on the microfiber.^24^ Finally, the selected aptamers (NE-AP and LF-AP) were respectively incubated onto the functional regions of two tapered optical fibers for 2 h. Then the excess proteins were washed away with deionized water. Incubation for too long a time may lead to protein accumulation, which reduces the binding rate between the aptamers and the target proteins, thus affecting the performance of the sensor. Meanwhile, the schematic diagram of the sensor preparation was presented (Figure S2).

It is acknowledged that bare tapered optical fibers are relatively fragile in terms of mechanical strength. To ensure the robustness of the sensing platform, the fabricated tapered fiber sensor in this study was integrated and packaged into a customized microfluidic chip channel. This design provides solid physical support and protection for the fiber, effectively preventing accidental bending or damage during handling, while enabling precise sample introduction and minimal volume consumption.

To verify reproducibility, six independent sensors were fabricated and tested with standard refractive index solutions. The results showed that the relative standard deviation (RSD) of their sensitivity values was less than 5%, demonstrating good fabrication repeatability. Furthermore, repeated measurements of the same target concentration using a single sensor exhibited high response consistency (RSD <3%).

### Quantification and statistical analysis

All spectral data acquisition and wavelength shift analysis were performed using the AQ6370E spectral analyzer (YOKOGAWA, Japan) with Origin 2020 (OriginLab Corporation, USA). SPR sensorgrams were analyzed using BIA Evaluation Software version 3.2 (BIAcore AB, Sweden). Statistical analyses and graphical presentations were conducted using GraphPad Prism version 10.0 (GraphPad Software, USA) and Origin 2020. Image densitometry was performed using ImageJ (USA National Institutes of Health). Molecular docking and structural predictions were visualized using BioGDP, NUPACK, RNAComposer, PDBePISA, and PyMOL.

All experiments were performed with a minimum of three independent biological replicates unless otherwise specified. All quantitative data are presented as mean ± SD unless otherwise specified. Wavelength shifts (Δλ) are reported as mean ± SD from independent sensor measurements. SPR-derived K_d_ values are reported as mean ± SD from three independent determinations. Clinical characteristics in Table 1 are presented as mean ± SD. Calibration curve linearity (R^2^) and sensitivity (slope, nm/log M) were derived from linear regression. When data did not conform to normal distribution, results are additionally presented as median with IQR. For two-group comparisons, the two-tailed unpaired Student’s ∗t∗-test was applied when data met normality and homogeneity of variances; otherwise, the Mann–Whitney U test was used. Temporal changes across multiple time points (Figures 5C and 5E) were analyzed using one-way ANOVA with Tukey’s post hoc test. The relationship between Δλ and log-transformed protein concentration was fitted by linear regression (least-squares method); the linear dynamic range was 3.57 × 10^−20^ M to 3.57 × 10^−12^ M for ELANE and 1.28 × 10^−19^ M to 1.28 × 10^−12^ M for LF. For selectivity assessment (Figures 4G and 4H), the absence of significant wavelength shifts relative to baseline was used as the criterion for specificity.

Mice were randomly assigned to experimental groups using a computer-generated sequence; clinical samples were randomized and coded prior to measurement. All data acquisition and analysis were performed blinded to group allocation; for clinical samples, operators were blinded to clinical status until raw data were recorded, with decoding performed after all measurements were completed. No formal power calculation was performed; sample sizes were based on our previous works and standard practices. Patients with confirmed sepsis (Sepsis-3 criteria) were included; healthy controls had no acute or chronic inflammation. All LPS-injected mice surviving to designated time points were included. No data were excluded unless specified in figure legends. In case of sensor failure (fiber breakage or spectral instability), measurements were excluded and repeated. All successfully collected data were included without outlier exclusion. Clinical blood samples were processed within 2 h of collection; healthy serum samples were used as quality controls to monitor batch-to-batch consistency. Normality was assessed using Shapiro–Wilk test (α = 0.05); homogeneity of variances was assessed using Levene’s test (α = 0.05); non-parametric alternatives were applied when assumptions were violated. Residuals from linear regressions were checked using Q–Q plots and residual plots. Statistical significance was defined as two-tailed ∗p∗ <0.05, with notation: ns (∗p∗ ≥0.05), ∗ (∗p∗ <0.05), ∗∗ (∗p∗ <0.01), ∗∗∗ (∗p∗ <0.001), and ∗∗∗∗ (∗p∗ <0.0001). Exact ∗p∗-values are reported in figure legends and supplementary tables. The specific statistical test for each experiment is indicated in the corresponding figure legend.
