# Supplementary material for: Graphene-oxide-microfiber aptasensor for dynamic neutrophil monitoring: Bridging cytokine release syndrome and point-of-care sepsis diagnosis
Source: iScience. 2026 Oct 12;29(11):117823. doi: 10.1016/j.isci.2026.117823 (PMC13682345; doi:10.1016/j.isci.2026.117823)
Supplement: Supplementary file 1 [file mmc1.pdf]

iScience, Volume *29*

Supplemental information

Graphene-oxide-microfiber aptasensor for dynamic neutrophil monitoring: Bridging cytokine release syndrome and point-of-care sepsis diagnosis

Zhiheng Yu, Heshuang Dong, Xiaolin Chen, Mengjiang Xu, Ren Liu, Jingjing Wang, Cunliang Yang, Ke Wang, Xianyuan Zhao, Hao Jia, and Jijun Feng

Supporting Information

SUPPLEMENTARY FIGURES

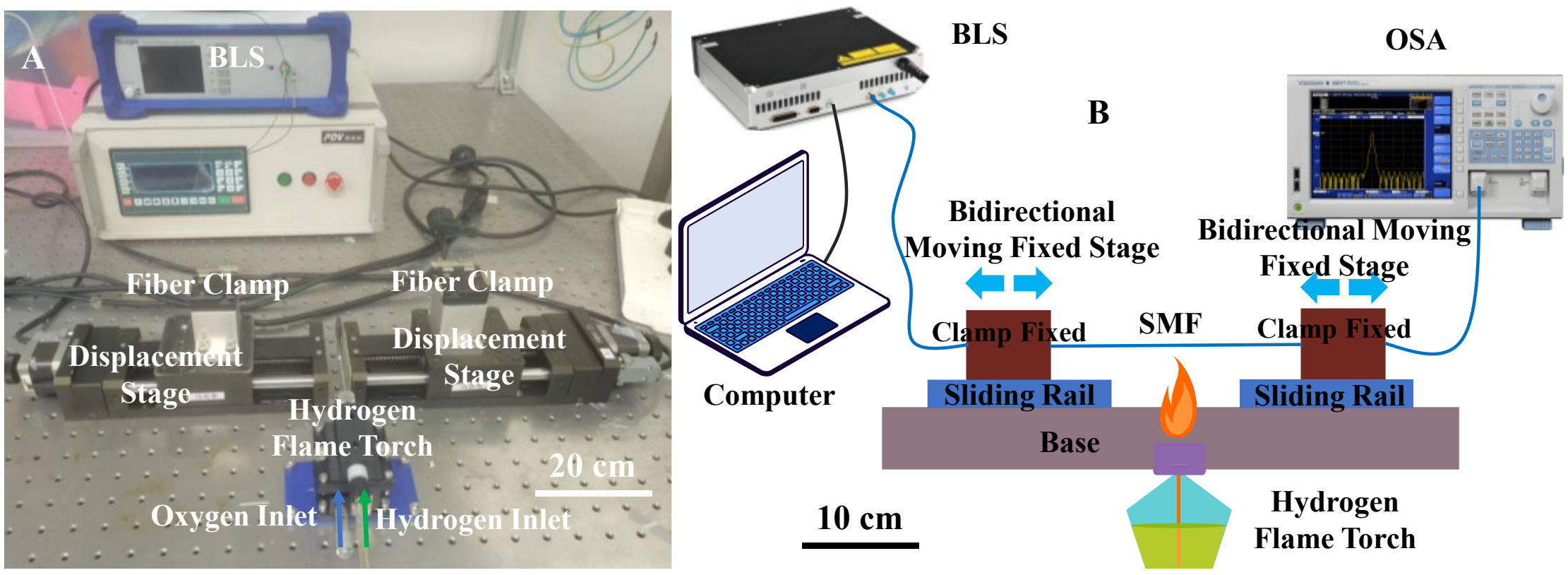

Figure S1. Fabrication of tapered optical fiber, related to STAR Methods.

(A) Physical picture of tapered optical fiber fabrication setup. Scale bars, 20 cm.

(B) Schematic diagram of tapered optical fiber fabrication. Scale bars, 10 cm.

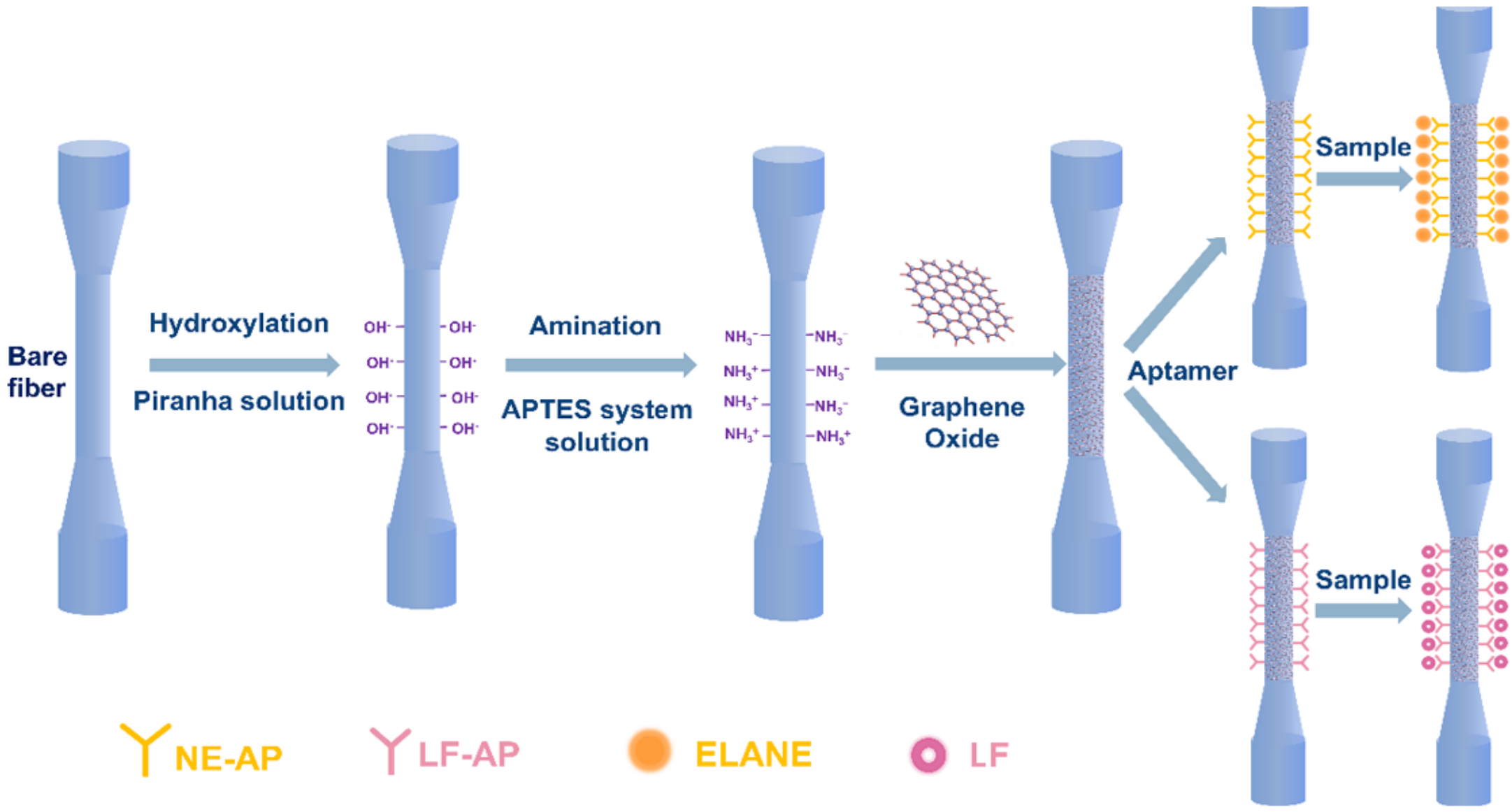

Figure S2. Schematic of the functionalization of microfiber sensor system, related to STAR Methods.

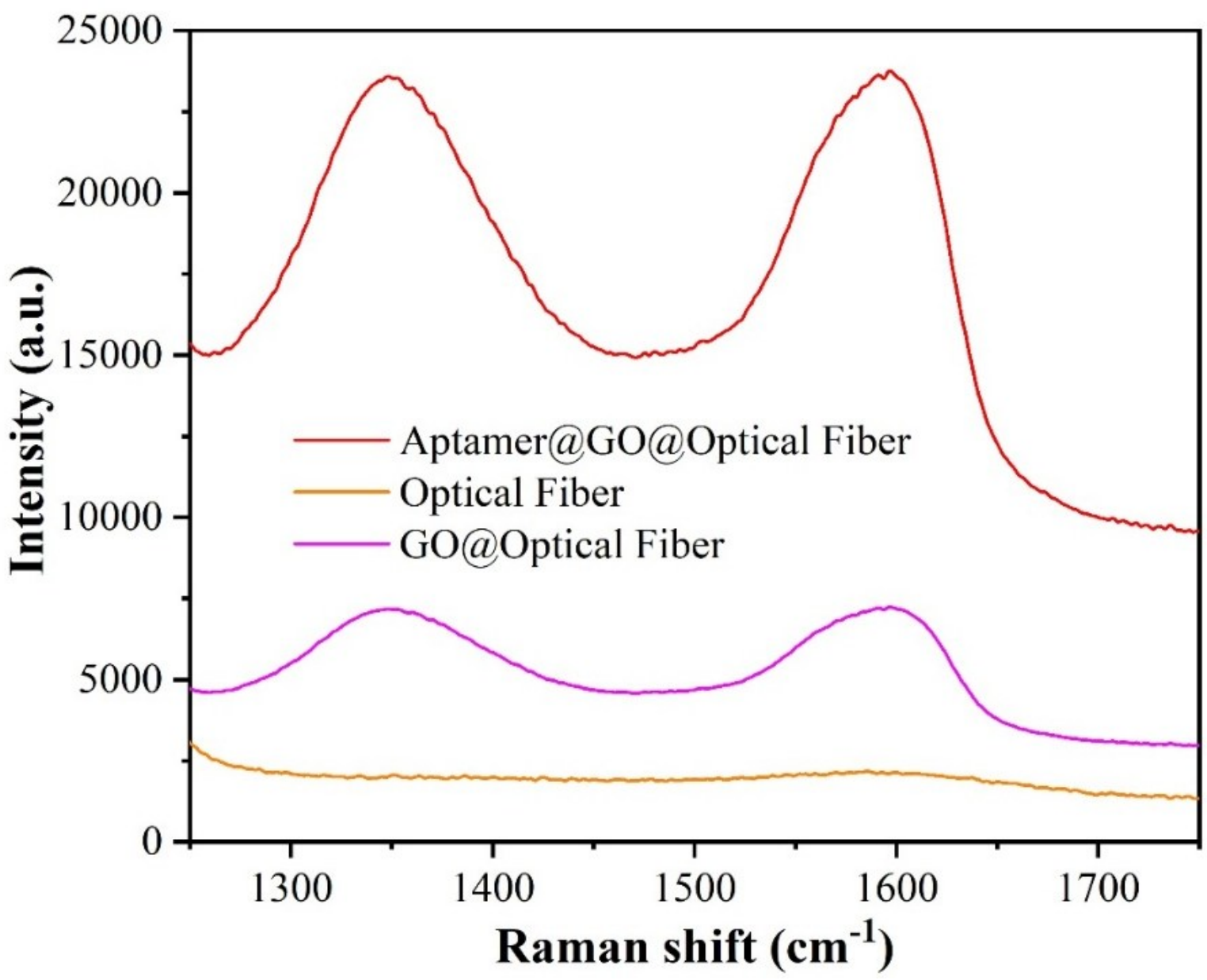

Figure S3. Raman spectrogram of Microfiber surface, related to Figure 3.

SUPPLEMENTARY TABLE

Table S1. Performance Comparison of Sensor Fabricated with the Conventional Methods

Method	LOD for ELANE/M	LOD for LF/M	Detection Time/min	Ref
ELISA	/	1.7×10^{-9}	/	1
Electrochemistry	/	3×10^{-10}	/	2
ELISA	4.5×10^{-13}	/	/	3
In this work	3.57×10^{-20}	1.28×10^{-19}	3	

SUPPLEMENTARY METHODS

Methods S1: The Nucleotide Sequences of the LF-AP and NE-AP Aptamers

(1) The nucleotide sequences of the LF-AP:

AGGCAGGACACCGTAACCGGTGCATCTATGGCTACTAGCTTTTCCTGCCT

(2) The nucleotide sequences of the NE-AP

CACGATGGTTAGGCGGGCCTTGAGGCTAATAATGTTGTTA

Methods S2: The method used to calculate the LOD

The LOD was calculated using the 3σ/slope method according to IUPAC recommendations:

$$\mathrm{LOD} = 3 \times {\sigma_{\mathrm{blank}}}/{S}$$

Where: σ_{blank} is the standard deviation of the blank (ddH_2O) measurements; S is the slope of the calibration curve in the linear range.

SUPPLEMENTARY REFERENCES

1. Alhalwani, A. (2024). Development of an Indirect ELISA for the Detection of Lactoferrin in Type 2 Diabetes Plasma: A Novel Approach. Curr. Anal. Chem. 20, 209-216. https://www.eurekaselect.com/article/138406.
2. Rabbani, G., Khan, M.E., Zakri, W., Khan, M.V., Bashiri, A.H. (2024). An electrochemical immunosensor based on AgNPs/Nafion-GCE for detection of salivary lactoferrin: Alzheimer's disease biomarker. Microchem. J. 207, 112079. https://doi.org/10.1016/j.microc.2024.112079.
3. Adamiec-Mroczek, J., Kluz, J., Chwalek, S., Rabczynski, M., Gostomska-Pampuch, K., Lewandowski, L., Misiuk-Hojlo, M., Ponikowska, B., Chourasia, G., Dumas, I., Gamian, A., Fiodorenko-Dumas, Z., Konopska, B., Gola, A., Konikowska, K., Strub, D., Bronowicka-Szydelko, A., Madziarska, K. (2025). Development of an enzyme-linked immunosorbent assay (ELISA) for determining neutrophil elastase (NE) - a potential useful marker of multi-organ damage observed in COVID-19 and post-Covid-19 (PCS). Front. Mol. Biosci. 12, 1542898. https://doi.org/10.3389/fmolb.2025.1542898.
